# Decreased* Clostridium* Abundance after Electroconvulsive Therapy in the Gut Microbiota of a Patient with Schizophrenia

**DOI:** 10.1155/2019/4576842

**Published:** 2019-02-25

**Authors:** Misako Kanayama, Maiko Hayashida, Sadayuki Hashioka, Tsuyoshi Miyaoka, Masatoshi Inagaki

**Affiliations:** Department of Psychiatry, Shimane University of Medicine, 89-1 Enyacho, Izumo 6938501, Japan

## Abstract

Relationships between gut microbiota and various disease pathogeneses have been investigated, but those between the pathogeneses of mental illnesses, including schizophrenia, and gut microbiota have only recently attracted attention. We observed a change in the gut microbiota of a patient with schizophrenia after administering electroconvulsive therapy (ECT). A 59-year-old woman was diagnosed with schizophrenia at 17 years of age and has been taking antipsychotic drugs since the diagnosis.* Clostridium*, which occupied 86.5% of her bacterial flora, decreased to 72.5% after 14 ECT sessions, while* Lactobacillus* increased from 1.2% to 5.5%, and* Bacteroides* increased from 9.1% to 31.5%. Previous studies have shown that* Clostridium* spp. are increased in patients with schizophrenia compared with those in healthy individuals and that* Clostridium* is reduced after pharmacological treatment. Our report is the first report on the gut microbiota of a patient with schizophrenia receiving ECT. Our results indicate that studies focusing on* Clostridium* to clarify the pathogenesis of schizophrenia as well as potential therapeutic mechanisms may be beneficial. However, further studies are needed.

## 1. Introduction

Schizophrenia is a severe psychiatric disorder, but its pathophysiology and therapeutic mechanisms remain unclear. In recent years, gut microbiota's influence on various biological functions and disease pathogeneses [1–3], including those of schizophrenia [4–7], has been investigated. Many studies have illustrated that gut microbiota interacts with the immune system, regulates brain activity, and influences behavioral processes. However, findings on gut microbiota characteristics in patients with schizophrenia have been less consistent [5].

Several bacterial genera, including* Clostridium*,* Bacteroides*, and* Lactobacillus*, have attracted attention as research targets. Three previous studies found that* Clostridium* is more abundant in the gut microbiota of patients with schizophrenia compared with those of healthy individuals [8–10], although one study reported the opposite result [11]. One report demonstrated that* Clostridium* abundance declined in patients with schizophrenia after the patients received drug treatment [9]. However, no study has investigated the changes in the gut microbiota before and after electroconvulsive therapy (ECT) in patients with schizophrenia. Herein, we report a case of a patient with schizophrenia treated with ECT and the changes in her gut microbiota after ECT.

## 2. Case Presentation

A 59-year-old woman was diagnosed with schizophrenia at 17 years of age and had been taking antipsychotic drugs since the diagnosis. She had no history of alcohol consumption or drug abuse. At age 57, she was admitted to another mental hospital after entering a catatonic state that was resistant to several antipsychotic drugs and benzodiazepines. She experienced delusions, hallucinations, grossly disorganized and catatonic behavior, and negative symptoms lasting more than 6 months. She showed no symptoms of organ disease on blood tests, neurological tests, or magnetic resonance imaging (MRI) of the head. Thus, we diagnosed her with schizophrenia based on the Diagnostic and Statistical Manual of Mental Disorders, 5^th^ edition (DSM-5). At her one-year follow-up after ECT, she had no symptoms indicating organ disease, and her diagnosis remained the same. In addition, she exhibited stupor, catalepsy, waxy flexibility, negativism, mannerism, stereotypy, agitation not influenced by external stimuli, and echolalia. Thus, we diagnosed her with catatonia per the DSM-5. No EEG and CSF analysis had been performed. Therefore autoimmune NMDA-R-encephalitis and potentially associated viral infections (e.g., Herpes; Influenza) had not been sufficiently ruled out.

She had been taking 9 mg of risperidone daily for 2 years for her severe symptoms without exothermic reactions or increased creatine phosphokinase (CPK); thus, she continued taking risperidone for her severe symptoms. Clozapine could not be administered because her white blood cell count was too low. No other symptoms of cytopenia, infection, or blood disorders were observed. She had no symptoms of organ disease. We suspected that the antipsychotic drugs were affecting her blood cell counts, but we determined that the cell counts were not clinically significant. Her white blood cell counts did not change, even after receiving ECT.

In addition, she had been taking 1.2 g magnesium oxide and 36 mg sennoside daily for constipation for many years during and after ECT. Her laxative remained unchanged. We did not exclude organ digestive diseases using advanced methods such as endoscopy. Instead, we judged clinically that her constipation were due to adverse effects from the drugs and her lifestyle habits. She had no intestinal obstruction or diseases that caused outright autonomic nervous abnormalities.

The patient's feces were sampled 1 month after admission (on the day before administering ECT) and 2 days after the final ECT session. The feces were collected promptly after defecation and stored at below -20°C until analysis. The bacterial composition of the gut microbiota was analyzed using the terminal restriction fragment length polymorphism method (Techno Suruga Labo Co. Ltd., Shizuoka, Japan). Her psychiatric symptoms were also evaluated using the Brief Psychiatric Rating Scale (BPRS) and the Bush-Francis Catatonia Rating Scale (BFCRS) [12]. We assumed that she lacked the capacity to provide informed consent; therefore, her father provided written informed consent for both publishing and this protocols approved by the Ethics Committee of Shimane University Hospital (no. 20160727-1).

We performed 14 ECT sessions (three times per week) in accordance with the revised edition of the Electrical Convulsion Therapy (ECT) Recommendation of the Department of Neuropsychiatry of Japan, which was created by translating the American Psychological Association (APA) treatment guidelines. The number of ECT sessions was reevaluated at the 10th session and continued. We used thiopental or ketamine as an anesthetic and stopped her breakfast on the morning of the ECT. After ECT, her BPRS score decreased from 83 to 65, and her BFCRS score decreased from 51 to 28. In addition, her gut microbiota differed before and after ECT.* Clostridium* decreased from 86.5% to 72.5%, while* Lactobacillus* increased from 1.2% to 5.5%, and* Bacteroides* increased from 9.1% to 31.5%.

## 3. Discussion

In this case, we observed a decrease in the* Clostridium* abundance and increases in the* Bacteroides* and* Lactobacillus* abundances after ECT. Thus, compared with previous pharmacological studies on gut microbiota, both ECT and antipsychotic drug treatments appear to decrease the amount of* Clostridium* in the gut microbiota of patients with schizophrenia. At least three studies [8–10] have reported that* Clostridium* was more abundant in the gut microbiota of patients with schizophrenia than in those of healthy individuals. Our report is the first to report the gut microbiota of a patient with schizophrenia receiving ECT. Our results were consistent with those of Yuan et al. (2018), who published the only study to examine changes in the gut microbiota before and after a 24-week risperidone treatment in patients with schizophrenia [9].

We also observed that* Lactobacillus* and* Bacteroides* increased after ECT. However, no consensus exists on the differences in these floras among healthy individuals or how they change after antipsychotic treatment [9–11].

Given the consistent results regarding the reduction in* Clostridium* after both pharmacotherapy and ECT, we considered three possibilities. First, ECT ameliorated the psychiatric symptoms, and the* Clostridium* abundance decreased because of the psychiatric symptom relief. Second, ECT ameliorated the psychiatric symptoms and independently reduced the* Clostridium* abundance. Third, ECT directly reduced the* Clostridium* abundance by altering the autonomic nervous system and/or immune system, and this reduction in* Clostridium* ameliorated the psychiatric symptoms.

Though there is no definite consensus, we are interested in the third hypothesis. Several studies support the third hypothesis that the mechanisms of the ECT's therapeutic effects are related to the autonomic nervous [13, 14] and immune systems [15–17]. Several studies have also reported that the gut microbiota may influence both the autonomic nervous and immune systems by increased intestinal permeability, an intestinal catecholamine receptor, or various other phenomena such as antigen-antibody reactions [4–6, 18, 19]. Investigations of these hypotheses might help clarify schizophrenia's pathophysiology and therapeutic mechanisms, for example, via immunological, autonomic, or other mechanisms or pathophysiologies; thus, future research is needed.

This case report has some limitations. First, the patient's diet may have influenced the results. However, her diet changed only slightly from the time of her hospital admission at age 55 to the final ECT sessions. Her eating [20, 21], bowel, and exercise [22] habits remained the same before and after ECT based on her medical records regarding diet contents and amount, time and form of defecation, and amount of activity during the day, excluding that she did not eat breakfast on the mornings she had ECT. In addition, some reports suggest that the variation in gut microbiota due to diet is relatively small [20, 23]. However, the influence of the diet remains unclear, and more research is needed.

Second, the drugs that the patient was taking may have affected the results. However, her medication details remained the same before and after ECT (for two years). In addition, as for anesthesia, how the anesthetic affects gut microbiota is unknown. In this case, since no convulsions occurred with the first anesthetic used, the physician changed the anesthetic to ketamine. In Japan, propofol is used as the first anesthetic, but this drug increases the seizure threshold. In cases where high seizure thresholds are a problem, ketamine is used because it does not raise the seizure threshold. The patient had no depressive symptoms. Although ketamine may affect gut microbiota, such as* Lactobacillus* [24], the effects of anesthesia on the gut microbiota were unclear in this case. Further research is necessary. Thus, whether the observed changes in the gut microbiota are causally linked to ECT or whether they occur by chance or other phenomena remains unclear.

Third, repeated fecal measurements may have been more informative. However, taking more fecal samples was not possible, which may have been a limitation.

Because previous studies have reported that* Clostridium* abundance decreases after drug therapy, and because this case demonstrated a decrease in* Clostridium* after ECT,* Clostridium* might be related to schizophrenia's therapeutic mechanisms and/or pathophysiology. Since this was a case report, this should be clarified through further research.
